# Delayed ischemic stroke after PED placement for aneurysms: optimal duration of dual antiplatelet therapy and risk factors

**DOI:** 10.3389/fneur.2025.1561965

**Published:** 2025-04-22

**Authors:** Chao Wang, Yangyang Zhou, Ying Zhang, Dachao Wei, Mirzat Turhon, Jian Liu, Yisen Zhang, Kun Wang, Hongqi Zhang, Tianxiao Li, Aisha Maimaitili, Guohua Mao, Donglei Song, Yunyan Wang, Wenfeng Feng, Yang Wang, Huaizhang Shi, Jieqing Wan, Jianmin Liu, Sheng Guan, Yuanli Zhao, Wenqiang Li, Xinjian Yang

**Affiliations:** ^1^Department of Interventional Neuroradiology, Beijing Neurosurgical Institute, Capital Medical University, Beijing, China; ^2^Department of Neurosurgery, Beijing Tiantan Hospital, Capital Medical University, Beijing, China; ^3^Department of Neurosurgery, Xuanwu Hospital, Capital Medical University, Beijing, China; ^4^Department of Neurosurgery, Zhengzhou University People′s Hospital, Zhengzhou, China; ^5^Department of Neurosurgery, First Affiliated Hospital of Xinjiang Medical University, Urumqi, China; ^6^Department of Neurosurgery, Second Affiliated Hospital of Nanchang University, Nanchang, China; ^7^Department of Neurosurgery, Shanghai Donglei Brain Hospital, Shanghai, China; ^8^Department of Neurosurgery, Qilu Hospital of Shandong University, Jinan, China; ^9^Department of Neurosurgery, Nanfan Hospital, Guangzhou, China; ^10^Department of Neurosurgery, First Affiliated Hospital of Nanchang University, Nanchang, China; ^11^Department of Neurosurgery, First Affiliated Hospital of Harbin Medical University, Harbin, China; ^12^Department of Neurosurgery, Renji Hospital, School of Medicine, Shanghai Jiao Tong University, Shanghai, China; ^13^Department of Neurosurgery, Changhai Hospital Affiliated to Naval Medical University, Shanghai, China; ^14^Department of Neurointervention Radiology, First Affiliated Hospital of Zhengzhou University, Zhengzhou, China; ^15^Department of Neurosurgery, Peking University International Hospital, Beijing, China

**Keywords:** flow diverter, intracranial aneurysm, dual antiplatelet therapy, delayed ischemic stroke, rare complication

## Abstract

**Background:**

Delayed ischemic stroke (DIS) is a rare complication that may occur in patients with cerebral aneurysms treated with the Pipeline Embolization device (PED). This study aims to evaluate the characteristics of DIS following PED placement and to investigate the optimal duration of dual antiplatelet therapy (DAPT) in relation to the incidence of DIS.

**Methods:**

We conducted a multicenter retrospective cohort study on consecutive cases of intracranial aneurysms treated with PED. Patients were divided into two groups based on the timing of DAPT switching to monotherapy: early (<6 months) and late (≥6 months). To adjust for potential biases between the groups, inverse probability of treatment weighting (IPTW) was applied. Kaplan–Meier survival analysis and multivariate Cox regression were used to calculate cumulative DIS rates, and risk factors for DIS.

**Results:**

A total of 1,146 consecutive patients with 1,296 aneurysms were included, of whom 12 (0.96%) who received PED developed DIS. The late-switch group had a lower DIS rate compared to the early-switch group [0.5% (4 of 752 patients) vs. 2.0% (8 of 394 patients), *p* = 0.018], even after IPTW. Hypertension [hazard ratio (HR) 3.47, 95% CI: 1.045–11.552] and complete occlusion immediately post-procedure (HR 5.48, 95% CI: 3.048–9.868) were significant risk factors for DIS.

**Conclusion:**

DIS is a rare complication among patients treated with PED for cerebral aneurysms. Extending the duration of DAPT to at least six months may safer for the patients with hypertension and immediate complete occlusion.

## Introduction

Flow diverters, particularly the Pipeline embolization device (PED), have demonstrated significant advantages in the treatment of intracranial aneurysms. Initially, following Food and Drug Administration approval, PED was primarily used for large or complex aneurysms in the internal carotid artery (1, 2). However, with advancements in technology and growing clinical experience, its use has expanded to include a broader range of intracranial aneurysms, such as small aneurysms, non-saccular aneurysms, and aneurysms in the posterior circulation (3–5).

However, complications related to PED treatment for various aneurysms cannot be overlooked (6). Delayed ischemic stroke (DIS), defined as an ischemic event occurring one-month post-procedure, is relatively rare, with reported incidence rates ranging from 1 to 5% in the literature (4, 7–12). Nevertheless, when DIS occurs, it is associated with poor clinical outcomes. The risk factors for DIS in patients treated with PED, especially regarding the optimal timing of antiplatelet therapy, remain unclear. Therefore, this study aims to investigate the incidence and potential risk factors of DIS in a large cohort of patients in China treated with PED for intracranial aneurysms. Additionally, the study explores the association between the duration of dual antiplatelet therapy (DAPT) and the occurrence of DIS.

## Methods

### Ethics approval

All procedures performed in this study involving human participants were in accordance with the ethical standards of the institutional and/or national research committee and with the 1964 Helsinki Declaration and its later amendments or comparable ethical standards. The study protocol was reviewed and approved by the ethics committee of Beijing Tiantan Hospital, and the approval number given by the ethical board was KY 2018–098-02. And the requirement for written informed consent was waived by retrospective study.

### Data selection

This study drew on data from the post-market, multicenter, retrospective registry study of intracranial aneurysm embolization using the Pipeline Embolization Device (PED) in China (PLUS; ClinicalTrials.gov Identifier: NCT03831672). The PLUS study provides a comprehensive, real-world cohort to evaluate the safety and effectiveness of PED in treating intracranial aneurysms within the Chinese population.

Clinical and angiographic data were collected from the PLUS study, including patient characteristics (sex, age, and comorbidities such as hypertension, dyslipidemia, diabetes, previous stroke, and coronary heart disease), aneurysm characteristics (location, shape, and maximum diameter), procedural details (PED type, size, and adjunctive coiling), and follow-up treatment with dual or monotherapy and its duration.

### Endovascular PED procedure

Treatment decisions, whether to use the PED alone or in combination with coiling, were made by neurosurgeons at each center. In cases where aneurysms were irregularly shaped, had a daughter sac, or a history of sentinel headaches, or where PED displacement or shortening could lead to incomplete coverage of the aneurysm neck, coils were added. Patients received DAPT both before and after the procedure. For unruptured aneurysm patients, aspirin (100 mg daily) and clopidogrel (75 mg daily) were administered preoperatively for at least 5 days. In cases where patients were identified as non-responders to clopidogrel, aspirin (100 mg daily) and ticagrelor (90 mg twice daily) were used. Doses were adjusted preoperatively based on platelet function testing. For patients with ruptured aneurysms, a loading dose of aspirin and clopidogrel (300 mg each) was administered before anesthesia. Postoperatively, DAPT continued for 3–6 months, followed by aspirin monotherapy for an additional 3–6 months or indefinitely, based on the treating surgeon’s experience. The switch from dual to monotherapy was classified into two groups: <6 months (early switching group) and ≥ 6 months (late switching group).

All embolization procedures were performed under general anesthesia with full procedural heparinization. A triaxial support system was used to access the aneurysm. The PED (either Classic or Flex) was delivered to the parent artery and deployed to cover the aneurysm neck using a Marksman or Phenom-27 microcatheter (Medtronic, Minneapolis, Minnesota, USA). If the device did not expand adequately, additional techniques, such as using wires, catheters, or balloon angioplasty, were employed. Coils were deployed through a second microcatheter in the aneurysm sac if adjunctive coiling was deemed necessary by the operator.

### DIS definition and evaluation

DIS was defined as a transient ischemic attack or stroke with evidence of infarction on magnetic resonance imaging (MRI) or computed tomography (CT), resulting in new neurological deficits in the vascular territory supplied by the vessel containing the PED, occurring more than one month after the procedure (13). Patients experiencing transient or persistent neurological symptoms, such as unilateral limb weakness, slurred speech, dizziness, blurred vision, or ataxia more than one month after the procedure, were evaluated to determine whether these complications were related to PED placement. The evaluation team, consisting of a neurointerventionist and a radiologist, each with at least 5 years of experience, reviewed the clinical and imaging data independently. In cases of disagreement, a more experienced neurointerventionist (with 10 years of experience) was consulted to make the final decision. Once the patient’s condition stabilized, cerebral angiographic imaging (digital subtraction angiography, CT angiography, and MR angiography) was performed to assess further treatment options. All patients were evaluated with regular follow-up over 1 year at least.

### Statistical analyses

Statistical analyses were conducted using the R Studio software package (version 4.1.2). Categorical variables were compared using the chi-squared (*χ*^2^) test or Fisher’s exact test, while continuous variables were analyzed with either the unpaired t-test or the Mann–Whitney U test. Patients were divided into two groups based on the duration of DAPT: <6 months (early group) and ≥ 6 months (late group). To adjust for baseline differences between the groups, inverse probability of treatment weighting (IPTW) was applied where the *p*-value was less than 0.05. Cumulative DIS rates in each group were calculated using Kaplan–Meier analysis and compared using log-rank tests. Variables with an unadjusted *p*-value of less than 0.1 in the bivariate analysis were included in a Cox proportional hazards model. A p-value of <0.05 was considered statistically significant.

## Results

### Baseline information

**Figure 1:**
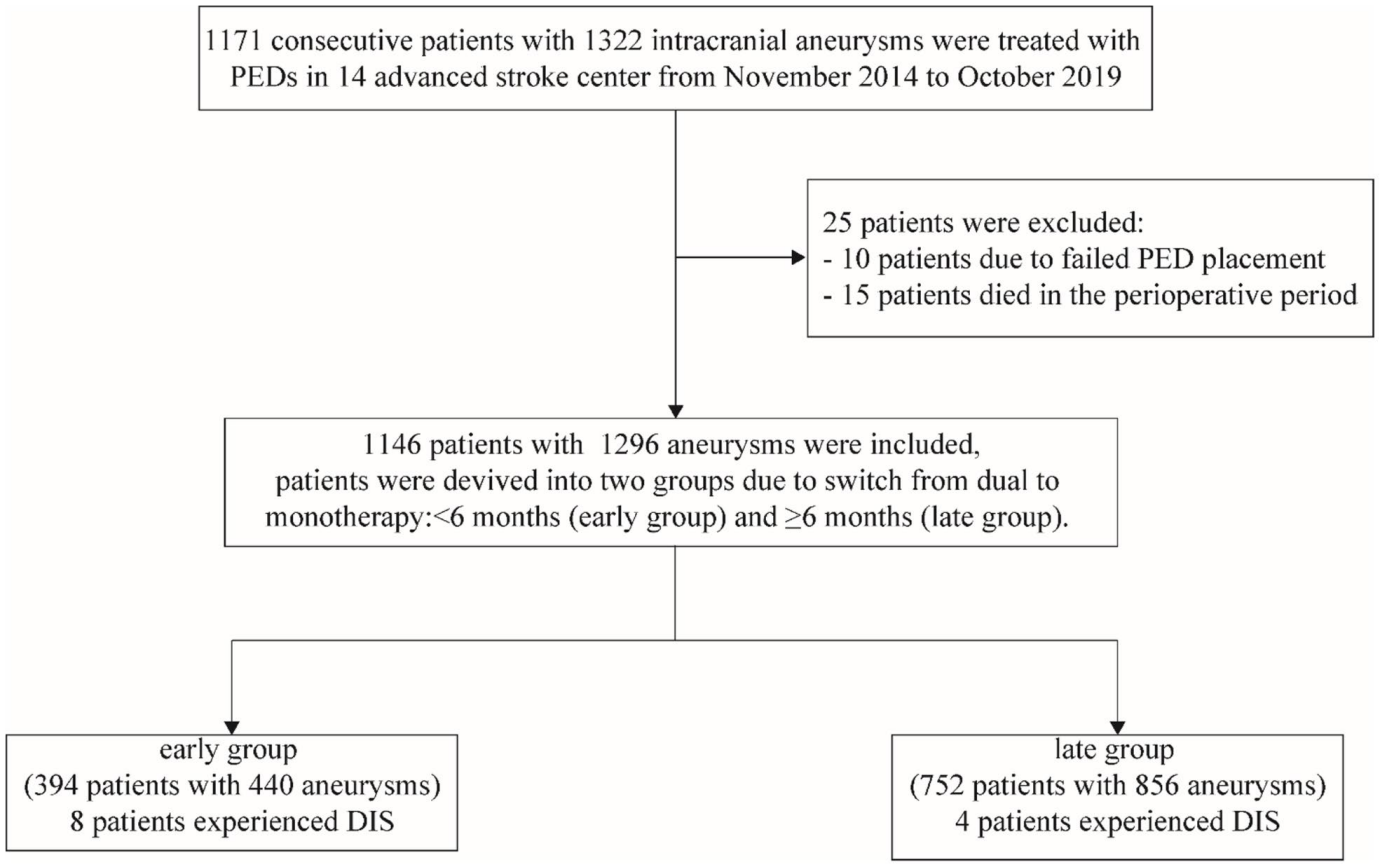
Flow chart for patients in this study. PED, pipeline embolization device; DIS, delayed ischemic stroke.

Between November 2014 and October 2019, a total of 1,171 patients with 1,322 aneurysms were treated with the Pipeline Embolization Device (PED) across 14 centers in China. After excluding 10 patients due to failed PED placement and 15 patients who died during the perioperative period (within 1 month), 1,146 patients with 1,296 aneurysms were included in the final analysis (flow chart in Figure 1).

### Incidence and outcomes of DIS

**Figure 2:**
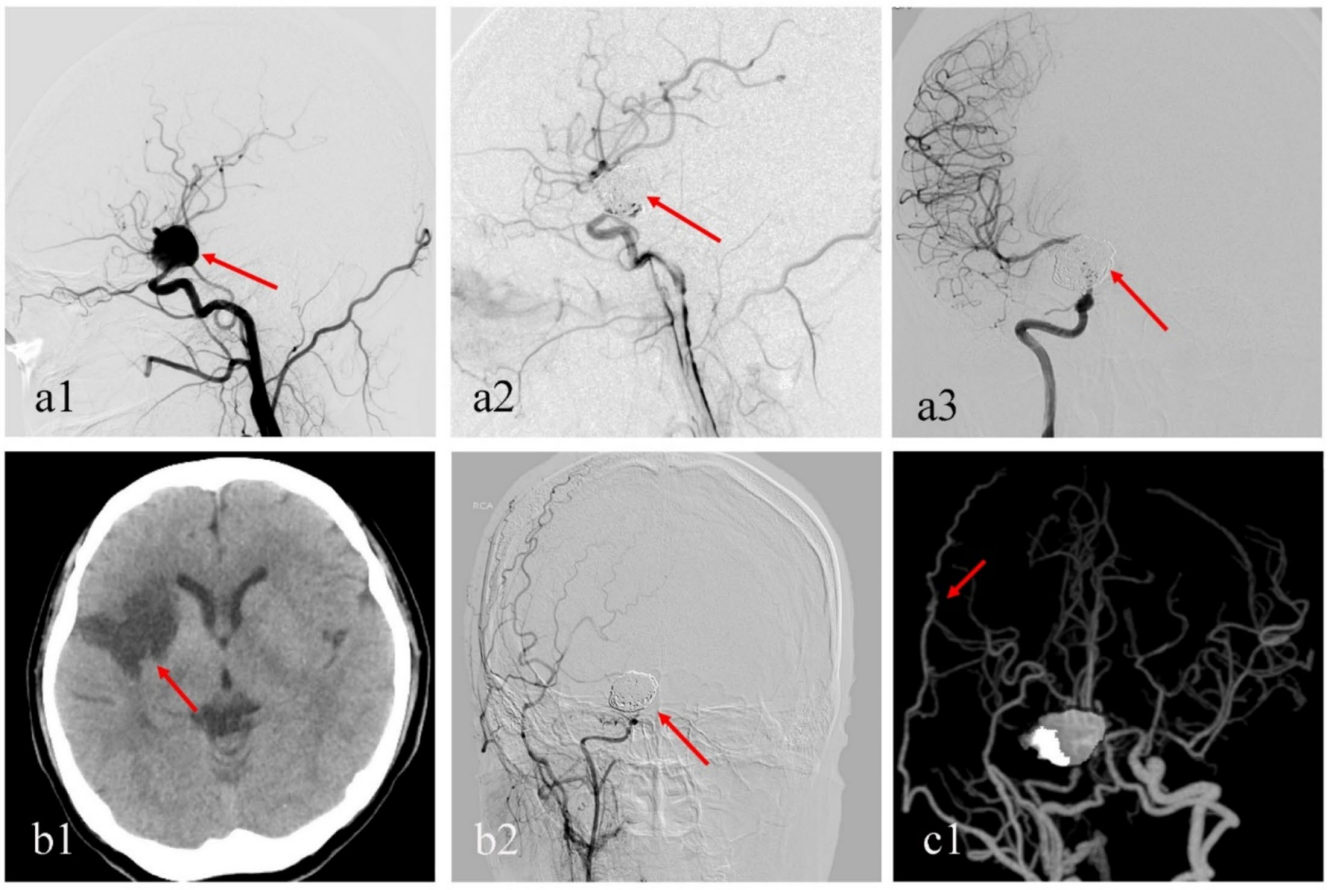
A female patient with a giant aneurysm at the distal end of the right internal carotid artery, approximately 25 mm in size **(a1)**. She underwent a PED (3.0*35 PED Classic) combined with coiling, and immediate postoperative angiography showed aneurysm occlusion **(a2)**. At the 3-month follow-up, the aneurysm was complete occlusion, but there was a suspicion of parent stenosis **(a3)**. Six months post-procedure, the dual antiplatelet therapy switching monotherapy. Ten months post-procedure, she experienced sudden headaches accompanied by projectile vomiting, facial asymmetry, and right-sided hemiplegia, with right cerebral infraction by CT **(b1)**. After rehabilitation, her neurologic function improved (modified Rankin Scale grade 2), but she continues to experience intermittent occurrences of transient ischemic attacks. Follow-up cerebral angiography showed a narrowing at the distal end of the internal carotid artery and the middle cerebral artery **(b2)**. She subsequently underwent extracranial-intracranial bypass surgery and has now fully recovered **(c1)**.

**Figure 3:**
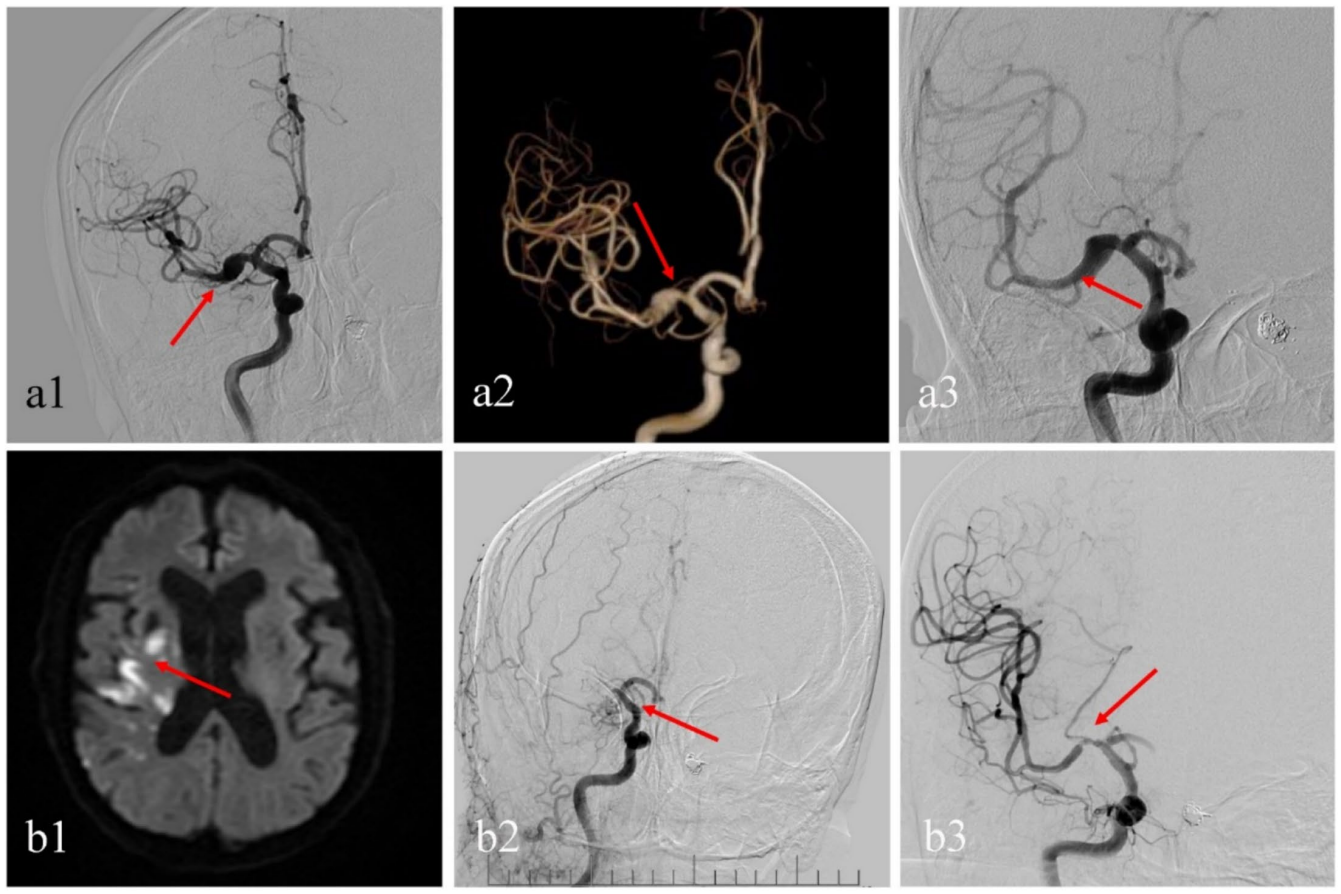
A male patient diagnosed with a right middle cerebral artery dissecting aneurysm **(a1,a2)**. He received treatment with a single PED (3.0*25 PED flex). Postoperative angiography immediately showed good adherence of the PED **(a3)**. However, four months post-procedure, the patient switched dual antiplatelet therapy to monotherapy. Five months post-procedure, he suddenly developed left-sided limb weakness and speech difficulties. Upon emergency admission, multiple infarctions were found on the right cerebral side **(b1)**. Emergency cerebral angiography and mechanical thrombectomy (aspiration thrombectomy) was performed, and postoperatively, the middle cerebral artery aneurysm was occlusion, but PED existed moderate stenosis **(b2,b3)**. Upon discharge, the patient’s Modified Rankin Scale score was 3.

A total of 12 patients experienced delayed ischemic stroke (DIS) between 3 and 16 months post-procedure, resulting in an incidence rate of 0.96%. Of these, five patients suffered transient ischemic attacks, and seven experienced infarctions. Imaging revealed that the embolized area corresponded to the location where the PED had been deployed, with no additional infarcts detected. The mean age of these patients was 54.8 years (range 35–75 years), and 8 of the 12 patients were female. Ten aneurysms were located in the proximal internal carotid artery (ICA), one in the middle cerebral artery, and one in the V4 segment of the vertebral artery. Additionally, four aneurysms were non-saccular, and three were classified as giant aneurysms (maximum diameter ≥ 25 mm). Notably, only one patient experienced DIS while on DAPT. Three patients suffered strokes after discontinuing antiplatelet agents: one patient discontinued the medication independently 3 months after the procedure, while the other two discontinued it based on the recommendations of their respective interventionalists 6 months post-procedure. The remaining patients experienced strokes during the monotherapy period (Table 1). Two patients experienced strokes due to stenosis or occlusion of the parent artery covered by the PED: one involving a non-saccular aneurysm of the middle cerebral artery and the other a terminal segment ICA. They underwent aspiration thrombectomy and bypass surgery; respectively (Figures 2, 3). The other patients received medical treatment and rehabilitation, with only one patient exhibiting poor neurological function (modified Rankin Scale score = 3) at the last follow-up. No major bleeding events, including intracranial or visceral hemorrhages, were observed during the follow-up period.

**Table 1:** Summary of patients who experienced DIS during follow-up after PED placement.

Case	Aneurysm location	Treatment strategy	DAPT time (months)	SAPT time (months)	Ischemic event time (months)	Follow up mRS score	Coexisting risk factor
1	Right ICA ophthalmic artery	Add coiling	6	Lifelong	4	0	Hypertension COAP
2	Left ICA ophthalmic artery	Add coiling	6	Lifelong	12	1	Hypertension
3	Right ICA terminus	Add coiling	6	12	10	0	COAP
4	Left ICA ophthalmic artery	PED alone	3	Lifelong	3	1	Hypertension
5	Right vertebral intracranial segment	Add coiling	6	Lifelong	8	1	-
6	Right ICA ophthalmic artery	Add coiling	3	Lifelong	16	0	COAP
7	Middle cerebral artery M1	PED alone	4	Lifelong	5	3	Hypertension
8	Left supraclinoid carotid artery	PED alone	3	Lifelong	7	1	Hypertension
9	Left ICA ophthalmic artery	Add coiling	3	Lifelong	6	1	-
10	Right supraclinoid carotid artery	Add coiling	3	3	5	1	Hypertension
11	Left ICA cavernous segment	Add coiling	3	6	6	1	Hypertension
12	Right ICA cavernous segment	PED alone	3	6	6	2	Hypertension COAP

All patients were adult patient.

COAP, complete occlusion immediately after procedure; DAPT, dual antiplatelet therapy; DIS, delayed ischemic stroke; ICA, internal carotid artery; PED, pipeline embolization device; SAPT, single antiplatelet therapy.

### Comparison of DIS rates between early and late switching from DAPT to monotherapy

Patients were divided into two groups based on the timing of switching from DAPT to monotherapy: the early group (394 patients with 440 aneurysms) and the late group (752 patients with 856 aneurysms). Three baseline factors differed significantly between the groups. The early group had a higher prevalence of hypertension and cerebral atherosclerosis compared to the late group [153/394 (38.8%) vs. 235/752 (31.2%), *p* = 0.012; 80/394 (20.3%) vs. 90/752 (12.0%), *p* < 0.001]. Additionally, PED combined with coiling was more frequently used in the early group [235/440 (53.4%) vs. 394/856 (46.0%), *p* = 0.014] (Table 2).

**Table 2:** Baseline characteristics of the subgroups, stratified by the time point of switching DAPT to monotherapy from PEDs placement before and after applying IPTW, after excluding patients who the death during perioperative and PED placement failure (25 patients with 26 aneurysms).

PED subgroups before IPTW					PED subgroups after IPTW		
Parameter	Total	<6 months DAPT subgroup	≥6 months DAPT subgroup	*p* value	<6 months DAPT subgroup	≥6 months DAPT subgroup	*p* value
No. of female (1146) (%)	779 (69.7)	267 (67.8)	532 (70.7)	0.330	272.5 (69.2)	529.0 (70.3)	0.702
Mean age (y) ± SD	53.96 ± 11.24	54.72 ± 11.05	53.57 ± 11.32	0.099	54.3 (11.2)	53.7 (11.2)	0.373
aSAH history (%)*	38 (3.3)	18 (4.6)	20 (2.7)	0.123	16.5 (4.2)	20.3 (2.7)	0.171
Co-morbidities							
Hypertension (%)	388 (33.7)	153 (38.8)	235 (31.2)	0.012	134.2 (34.1)	255.6 (34.0)	0.968
Diabetes mellitus (%)	61 (5.3)	27 (6.9)	34 (4.5)	0.126	26.3 (6.7)	35.4 (4.7)	0.167
Hyperlipidemia (%)	42 (3.8)	14 (3.6)	28 (3.7)	1.0	13.4 (3.4)	30.0 (4.0)	0.615
Cerebral infarction (%)	53 (4.6)	24 (6.1)	29 (3.9)	0.118	22.2 (5.6)	31.9 (4.2)	0.297
Cerebral atherosclerosis (%)	170 (14.8)	80 (20.3)	90 (12.0)	<0.001	88.9 (22.6)	171.1 (22.7)	0.953
Coronary heart disease (%)	58 (5.1%)	23 (5.8)	35 (4.7)	0.468	20.4 (5.2)	36.6 (4.9)	0.819
Alcohol abuse (%)	141 (12.3)	50 (12.7)	91 (12.1)	0.846	47.6 (12.1)	93.0 (12.4)	0.889
Smoking (%)	310 (29.7)	93 (23.6)	207 (27.5)	0.173	89.8 (22.8)	209.9 (27.9)	0.068
Aneurysm location (1296) (%)				0.643			0.134
Anterior circulation	1,130 (87.2)	381 (86.6)	749 (87.5)		384.1 (87.5)	723.3 (84.4)	
Posterior circulation	166 (12.8)	59 (12.3)	107 (12.5)		54.7 (12.5)	133.9 (15.6)	
Mean aneurysm diameter (mm) ± SD	11.7 ± 8.7	12.3 ± 8.8	11.5 ± 8.6	0.092	13.1 (8.9)	12.2 (8.6)	0.114
< 10 mm (%)	663 (51.2)	215 (48.9)	448 (52.3)	0.298	213.6 (48.7)	452.5 (52.9)	0.165
10–25 mm (%)	514 (39.7)	178 (40.5)	336 (39.3)		177.8 (40.5)	336.4 (39.2)	
> 25 mm (%)	119 (9.2)	47 (10.7)	72 (8.4)		47.4 (10.7)	68.3 (7.9)	
Non- saccular (%)	221 (17.1)	71 (16.1)	150 (17.5)	0.582	70.6 (16.2)	147.8 (17.3)	0.618
PED flex (1290) (%)	701 (54.3)	234 (53.4)	467 (54.8)	0.636	224.3 (51.3)	459.6 (53.9)	0.363
Combined with coiling (%)	629 (48.5)	235 (53.4)	394 (46.0)	0.014	225.2 (51.3)	405.3 (42.3)	0.173
PED size							
Device lengths	25.4 ± 5.6	25.0 ± 5.5	25.6 ± 5.7	0.113	25.0 (5.4)	25.6 (5.7)	0.087
Device widths	4.11 ± 0.53	4.1 ± 0.6	4.1 ± 0.5	0.359	4.1 (0.5)	4.1 (0.5)	0.432
Complete occlusion after PED treatment	190 (14.7)	64 (14.5)	126 (14.7)	0.999	63.3 (14.4)	126.8 (14.8)	0.822
Follow up (months)	21.3 ± 6.0	20.9 ± 7.8	21.5 ± 6.5	0.7167	20.3 ± 7.4	20.8 ± 6.1	0.222

IPTW, IPTW balancing of the three baseline factors: history of hypertension, cerebral atherosclerosis, and whether PED was combined with coiling.

aSAH, aneurysmal subarachnoid hemorrhage; IPTW, inverse probability of treatment weighting; PED, pipeline embolization device; DAPT, dual antiplatelet drug.

*The aSAH history indicate that the patients underwent aneurysmal subarachnoid hemorrhage before using PED treatment.

**Figure 4:**
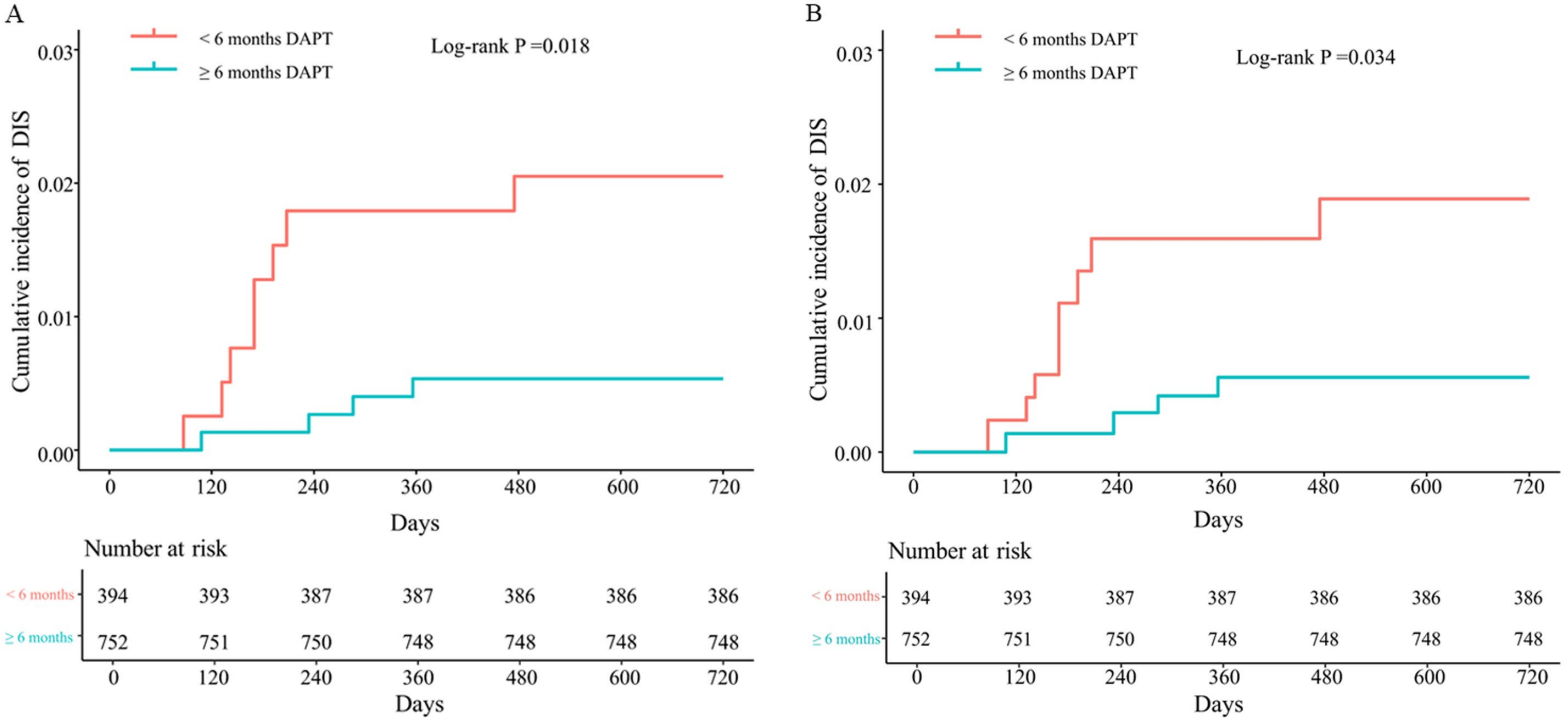
Kaplan–Meier curve shows cumulative rates of delayed ischemic stroke after PED placement for early versus later switch, stratified by duration 6 months to switch from dual antiplatelet therapy to monotherapy before **(A)** and after IPWT **(B)**.

The cumulative rates of DIS after switching to monotherapy were 2.0% (8/394) in the early group and 0.5% (4/752) in the late group (*p* = 0.018) (Figure 4A). After adjusting for the baseline differences using IPTW for hypertension, history of cerebral atherosclerosis, and PED combined with coiling, the cumulative rates of DIS remained significantly different: 1.9% (7.4/393.7) in the early group and 0.6% (4.2/752.3) in the late group (*p* = 0.034) (Figure 4B).

### Risk factors for DIS

We used the IPTW-adjusted data to analyze the risk factors for DIS. In univariate Cox regression analysis, the risk factors for DIS included hypertension (*p* = 0.04), giant aneurysm (maximum diameter ≥ 25 mm) (*p* = 0.054), classic PED deployment (*p* = 0.048), non-saccular aneurysms (*p* = 0.053), and complete occlusion immediately post-procedure (*p* = 0.009). In the Cox proportional hazards analysis, hypertension [hazard ratio (HR), 3.474; 95% confidence interval (CI): 1.045–11.55; *p* = 0.042] and complete occlusion immediately post-procedure (HR, 5.481; 95% CI: 3.048–9.868; *p* = 0.008) were identified as significant risk factors for DIS after PED placement (Table 3).

**Table 3:** Delayed ischemic stroke after PED deployed risk factors after IPTW.

Parameter	*P* value	HR (95% CI)
Hypertension	0.042	3.474 (1.045–11.552)
Complete occlusion immediately after procedure	0.008	5.481 (3.048–9.868)

## Discussion

We analyzed a large, multicenter, retrospective registry study in China to investigate the rate of DIS events and the associated risk factors in patients treated with the PED. Our findings indicate that while DIS is relatively rare in patients with aneurysms treated by PED, it can still result in permanent neurological deficits. We categorized patients into two groups based on the duration of DAPT: the late-switching group (≥6 months) and the early-switching group (<6 months). Our analysis revealed that delaying the switch from dual to monotherapy significantly reduced the rate of DIS, both before and after applying IPTW, which accounted for baseline differences between the groups. Additionally, hypertension and complete occlusion immediately post-procedure were identified as significant clinical risk factors for DIS.

### Impact of dual antiplatelet therapy duration on DIS events following flow diverter treatment

Numerous clinical studies have demonstrated that standard or platelet function-tailored DAPT reduce perioperative ischemic events in PED treatment for intracranial aneurysms (14–17). However, postoperative antiplatelet regimens are not standardized, and the optimal timing for switching from dual to monotherapy remains controversial, especially given the risk of DIS and bleeding complications (8).

For instance, the PUFS study included 108 patients with large or giant aneurysms who received dual antiplatelet therapy for 3 to 6 months. Four patients (3.7%, 4/108) developed delayed ischemic stroke (DIS) (1, 11). Guédon et al. collected data from 86 patients who underwent PED placement for intracranial aneurysms. These patients received dual antiplatelet therapy for 3 to 6 months post-procedure, followed by aspirin alone for 6 to 9 months. The study reported that 3 patients (3.5%, 3/86) experienced DIS at 4, 13, and 20 months, respectively (8). In the PREMIER study, only one patient experienced DIS after receiving 3 months of dual antiplatelet therapy (4). Hohenstatt et al. analyzed patients treated with the flow-redirection endoluminal device (FRED) with a follow-up of ≥5 years. Among them, 5 patients (7.4%, 5/68) experienced transient ischemic attacks (TIA) or cerebral infarctions. A thrombosis event occurred within 3 months despite dual antiplatelet therapy, while other four events occurred between 8 and 24 months after discontinuation of antiplatelet agents (10). A large study of the p64 Flow Modulation Device included 617 patients with anterior circulation aneurysms who received 12 months of dual antiplatelet therapy. Seven patients (1.1%, 7/617) developed DIS (in-stent thrombosis), which was attributed to discontinuation of dual antiplatelet therapy or drug interactions (7).

Although previous studies have reported the incidence of DIS and potential predisposing factors, they did not specifically address the timing of the switch from dual to monotherapy. Our study, which demonstrated a low DIS event rate (0.96%), is consistent with these findings. More importantly, we evaluated the optimal duration of DAPT for preventing DIS and found that longer DAPT duration significantly reduced the risk of DIS. The late-switching group had a lower incidence of DIS compared to the early-switching group, both before and after IPTW adjustment (2.0% vs. 0.5%, *p* = 0.018; 1.9% vs. 0.6%, *p* = 0.033). These results align with those of Hwang et al., who found that extending DAPT to 9 months reduced the incidence of DIS after stent-assisted coil placement (early vs. late switch, 5.6% vs. 0%, *p* = 0.013; midterm vs. late switch, 4.4% vs. 0%, *p* = 0.028) (18). However, it is important to note that while the Hwang study focused on stent-assisted coil placement, our study specifically investigated the use of PEDs.

### Risk factors for DIS

Cox regression analysis identified two significant risk factors for DIS: hypertension and complete occlusion immediately post-procedure. These findings are consistent with previous studies. For example, the DIVERSION prospective cohort study found that high baseline blood pressure was linked to an increased risk of neurological deficits (19). In our study, hypertension likely contributed to poorer parent artery conditions, which may have impaired proper PED deployment, leading to incomplete endothelialization at the aneurysm neck and an increased risk of thrombus dislodgement and cerebral infarction.

Complete occlusion immediately after the procedure, although generally considered favorable, may also increase the risk of DIS. Complete occlusion usually indicates dense packing of the aneurysm, but the PED acts as a flow diverter, gradually reducing blood flow into the aneurysm and promoting thrombosis. Dense packing, while effective at reducing aneurysm rupture (20), may cause incomplete device apposition, leading to thromboembolic events. This has been supported by other studies, such as Siddiqui et al., who reported that dense packing combined with PED for giant intracranial aneurysms increased thrombotic events and worsened mass effects (21).

### Safety and efficacy of prolonged dual antiplatelet therapy following PED treatment

Previous studies have categorized bleeding complications as major, minor, or minimal. Most bleeding events were classified as minor or minimal, and delayed aneurysm rupture was rarely reported during follow-up (22, 23). Our study found no major bleeding events, including intracranial or visceral hemorrhages, during the follow-up period, supporting the safety of extended DAPT beyond six months. In our study, 10 of 12 patients (83.3%) who experienced DIS had one or more risk factors. For these patients, prolonging DAPT may be beneficial in reducing the risk of DIS following PED treatment.

### Limitations

This study has several limitations inherent to its retrospective registry design. First, the duration of dual antiplatelet therapy (DAPT) within each switching group was not standardized across all patients. Patients were classified based solely on a 6-month DAPT period, although the duration of antiplatelet therapy ideally requires more precise definition. Second, the data were collected from multiple centers, which may have introduced variations in medical records and imaging quality, potentially leading to the omission of relevant information. Minor bleeding events, minimal bleeding events, and TIAs may therefore have been underreported. Third, not all patients underwent platelet function testing, making it impossible to evaluate the effect of antiplatelet resistance on DIS. However, DIS events were usually observed to occur after the transition from dual antiplatelet therapy to monotherapy. Furthermore, this study focused exclusively on the PED and did not include other flow diverters, such as the FRED, SURPASS, or P64. Additionally, the study focused only on the PED Classic and Flex, without considering the Pipeline Shield, a third-generation device widely adopted for treating intracranial aneurysms, which may impact antiplatelet regimens and therapy duration (24, 25).

## Conclusion

Our study analyzes the PLUS data and found 12 patients suffered from the DIS, account for 0.96% of total 1,146 patients. Compared to the <6 months dual antiplatelet switching time, ≥6 months switching time group had lower DIS occurrence rate. Besides, we analyze the risk factor of DIS, including hypertension history and aneurysm complete occlusion immediately after procedure. And it’s need to pay attention to the patients with above risk factors are prone to happened to DIS. Prolong the dual antiplatelet switching time at least 6 months for patients with risk factors might decrease the rate of DIS. But, due to the respective study, the evidence supporting this finding is limited. The delicate question of optimal DAPT duration should ideally be addressed through a prospective, randomized controlled trial.

## Data Availability

Publicly available datasets were analyzed in this study. This data can be found at: the data that support the findings of this study are available from the corresponding authors, upon reasonable request.

## References

[1] Becske T Kallmes DF Saatci I McDougall CG Szikora I Lanzino G. Pipeline for uncoilable or failed aneurysms: results from a multicenter clinical trial. Radiology. (2013) 267:858–68. doi: 10.1148/radiol.13120099, PMID: 23418004

[2] Deutschmann HA Wehrschuetz M Augustin M Niederkorn K Klein GE. Long-term follow-up after treatment of intracranial aneurysms with the pipeline embolization device: results from a single center. AJNR Am J Neuroradiol. (2012) 33:481–6. doi: 10.3174/ajnr.A2790, PMID: 22158922 PMC7966428

[3] Griessenauer CJ Enriquez-Marulanda A Taussky P Biswas A Grandhi R Xiang S. Experience with the pipeline embolization device for posterior circulations aneurysms: a multicenter cohort study. Neurosurgery. (2020) 87:1252–61. doi: 10.1093/neuros/nyaa277 32629474

[4] Hanel RA Kallmes DF Lopes DK Nelson PK Siddiqui A Jabbour P. Prospective study on embolization of intracranial aneurysms with the pipeline device: the premier study 1 year results. J Neurointerv Surg. (2020) 12:62–6. doi: 10.1136/neurintsurg-2019-015091, PMID: 31308197 PMC6996098

[5] Kiyofuji S Graffeo CS Perry A Murad MH Flemming KD Lanzino G. Meta-analysis of treatment outcomes of posterior circulation non-saccular aneurysms by flow diverters. J Neurointerv Surg. (2018) 10:493–9. doi: 10.1136/neurintsurg-2017-013312, PMID: 28965108

[6] Shehata MA Ibrahim MK Ghozy S Bilgin C Jabal MS Kadirvel R. Long-term outcomes of flow diversion for unruptured intracranial aneurysms: a systematic review and meta-analysis. J Neurointerv Surg. (2023) 15:898–902. doi: 10.1136/jnis-2022-019240, PMID: 36150896 PMC10033458

[7] Aguilar Pérez M Henkes E Hellstern V Serna Candel C Wendl C Bäzner H. Endovascular treatment of anterior circulation aneurysms with the p64 flow modulation device: mid- and long-term results in 617 aneurysms from a single center. Oper Neurosurg. (2021) 20:355–63. doi: 10.1093/ons/opaa425, PMID: 33469666 PMC8133326

[8] Guédon A Clarençon F Di Maria F Rosso C Biondi A Gabrieli J. Very late ischemic complications in flow-diverter stents: a retrospective analysis of a single-center series. J Neurosurg. (2016) 125:929–35. doi: 10.3171/2015.10.JNS15703, PMID: 26824382

[9] Hanel RA Cortez GM Lopes DK Nelson PK Siddiqui AH Jabbour P. Prospective study on embolization of intracranial aneurysms with the pipeline device (premier study): 3-year results with the application of a flow diverter specific occlusion classification. J Neurointerv Surg. (2023) 15:248–54. doi: 10.1136/neurintsurg-2021-018501, PMID: 35292570 PMC9985759

[10] Hohenstatt S Ulfert C Herweh C Hilgenfeld T Schmitt N Schönenberger S. Long-term follow-up after aneurysm treatment with the flow redirection endoluminal device (fred) flow diverter. Clin Neuroradiol. (2024) 34:181–8. doi: 10.1007/s00062-023-01346-3, PMID: 37833546 PMC10881684

[11] Becske T Brinjikji W Potts MB Kallmes DF Shapiro M Moran CJ. Long-term clinical and angiographic outcomes following pipeline embolization device treatment of complex internal carotid artery aneurysms: five-year results of the pipeline for uncoilable or failed aneurysms trial. Neurosurgery. (2017) 80:40–8. doi: 10.1093/neuros/nyw014 28362885

[12] Rossen JD Chalouhi N Wassef SN Thomas J Abel TJ Jabbour PM. Incidence of cerebral ischemic events after discontinuation of clopidogrel in patients with intracranial aneurysms treated with stent-assisted techniques. J Neurosurg. (2012) 117:929–33. doi: 10.3171/2012.8.JNS12185, PMID: 22957528

[13] Meyers PM Schumacher HC Higashida RT Derdeyn CP Nesbit GM Sacks D. Reporting standards for endovascular repair of saccular intracranial cerebral aneurysms. Stroke. (2009) 40:e366–79. doi: 10.1161/STROKEAHA.108.527572, PMID: 19246711

[14] Kim KS Fraser JF Grupke S Cook AM. Management of antiplatelet therapy in patients undergoing neuroendovascular procedures. J Neurosurg. (2018) 129:890–905. doi: 10.3171/2017.5.JNS162307, PMID: 29192856

[15] McTaggart RA Choudhri OA Marcellus ML Brennan T Steinberg GK Dodd RL. Use of thromboelastography to tailor dual-antiplatelet therapy in patients undergoing treatment of intracranial aneurysms with the pipeline embolization device. J Neurointerv Surg. (2015) 7:425–30. doi: 10.1136/neurintsurg-2013-011089, PMID: 24739599

[16] Saatci I Yavuz K Ozer C Geyik S Cekirge HS. Treatment of intracranial aneurysms using the pipeline flow-diverter embolization device: a single-center experience with long-term follow-up results. AJNR Am J Neuroradiol. (2012) 33:1436–46. doi: 10.3174/ajnr.A3246, PMID: 22821921 PMC7966552

[17] Hwang G Huh W Lee JS Villavicencio JB Villamor RBV Ahn SY. Standard vs modified antiplatelet preparation for preventing thromboembolic events in patients with high on-treatment platelet reactivity undergoing coil embolization for an unruptured intracranial aneurysm: a randomized clinical trial. JAMA Neurol. (2015) 72:764–72. doi: 10.1001/jamaneurol.2015.0654, PMID: 26010803

[18] Hwang G Kim JG Song KS Lee YJ Villavicencio JB Suroto NS. Delayed ischemic stroke after stent-assisted coil placement in cerebral aneurysm: characteristics and optimal duration of preventative dual antiplatelet therapy. Radiology. (2014) 273:194–201. doi: 10.1148/radiol.14140070 24918960

[19] Gory B Berge J Bonafé A Pierot L Spelle L Piotin M. Flow diverters for intracranial aneurysms. Stroke. (2019) 50:3471–80. doi: 10.1161/STROKEAHA.119.024722, PMID: 31765296

[20] Hassan T Ahmed YM Hassan AA. The adverse effects of flow-diverter stent-like devices on the flow pattern of saccular intracranial aneurysm models: computational fluid dynamics study. Acta Neurochir. (2011) 153:1633–40. doi: 10.1007/s00701-011-1055-9 21647821

[21] Siddiqui AH Kan P Abla AA Hopkins LN Levy EI. Complications after treatment with pipeline embolization for giant distal intracranial aneurysms with or without coil embolization. Neurosurgery. (2012) 71:E509–13. doi: 10.1227/NEU.0b013e318258e1f8, PMID: 22710418

[22] Mehran R Rao SV Bhatt DL Gibson CM Caixeta A Eikelboom J. Standardized bleeding definitions for cardiovascular clinical trials: a consensus report from the bleeding academic research consortium. Circulation. (2011) 123:2736–47. doi: 10.1161/CIRCULATIONAHA.110.009449, PMID: 21670242

[23] Theiss P Ali AE McGuire LS Lanzino G Ghozy S Brinjikji W. The natural history of aneurysms incompletely occluded by placement of a flow diverter: a multiinstitutional study. J Neurosurg. (2024) 141:310–5. doi: 10.3171/2023.12.JNS232221, PMID: 38457799

[24] Trivelato FP Wajnberg E Rezende MTS Ulhôa AC Piske RL Abud TG. Safety and effectiveness of the pipeline flex embolization device with shield Technology for the Treatment of intracranial aneurysms: midterm results from a multicenter study. Neurosurgery. (2020) 87:104–11. doi: 10.1093/neuros/nyz356, PMID: 31504821

[25] Martínez-Galdámez M Lamin SM Lagios KG Liebig T Ciceri EF Chapot R. Treatment of intracranial aneurysms using the pipeline flex embolization device with shield technology: angiographic and safety outcomes at 1-year follow-up. J Neurointerv Surg. (2019) 11:396–9. doi: 10.1136/neurintsurg-2018-014204, PMID: 30262655 PMC6582709

